# A novel lncRNA–protein interaction prediction method based on deep forest with cascade forest structure

**DOI:** 10.1038/s41598-021-98277-1

**Published:** 2021-09-23

**Authors:** Xiongfei Tian, Ling Shen, Zhenwu Wang, Liqian Zhou, Lihong Peng

**Affiliations:** School of Computer Science, Hunan University of Technology, Zhuzhou, 412007 China

**Keywords:** Computational biology and bioinformatics, Genetics, Systems biology, Mathematics and computing

## Abstract

Long noncoding RNAs (lncRNAs) regulate many biological processes by interacting with corresponding RNA-binding proteins. The identification of lncRNA–protein Interactions (LPIs) is significantly important to well characterize the biological functions and mechanisms of lncRNAs. Existing computational methods have been effectively applied to LPI prediction. However, the majority of them were evaluated only on one LPI dataset, thereby resulting in prediction bias. More importantly, part of models did not discover possible LPIs for new lncRNAs (or proteins). In addition, the prediction performance remains limited. To solve with the above problems, in this study, we develop a Deep Forest-based LPI prediction method (LPIDF). First, five LPI datasets are obtained and the corresponding sequence information of lncRNAs and proteins are collected. Second, features of lncRNAs and proteins are constructed based on four-nucleotide composition and BioSeq2vec with encoder-decoder structure, respectively. Finally, a deep forest model with cascade forest structure is developed to find new LPIs. We compare LPIDF with four classical association prediction models based on three fivefold cross validations on lncRNAs, proteins, and LPIs. LPIDF obtains better average AUCs of 0.9012, 0.6937 and 0.9457, and the best average AUPRs of 0.9022, 0.6860, and 0.9382, respectively, for the three CVs, significantly outperforming other methods. The results show that the lncRNA FTX may interact with the protein P35637 and needs further validation.

## Introduction

Noncoding RNAs regulate the majority of biological processes associated with development, differentiation, and metabolism in organisms^1^. In contrast to small noncoding RNAs (i.e., miRNAs), which are highly conserved and regulate transcriptional and posttranscriptional gene silencing^2,3^, long noncoding RNAs (lncRNAs), as one type of transcribed RNA molecules, are poorly conserved and control gene expression based on various mechanisms^4–6^. lncRNAs have close linkages with posttranscriptional gene regulation by regulating biological processes including protein synthesis, RNA maturation and transportation, and transcriptional gene silencing^7,8^. Although a few lncRNAs have been well studied, the biological functions of the majority of lncRNAs remain enigmatic^9^. Recent studies demonstrate that most of lncRNAs regulate various biological activities through specific associations with chromatin, for example, interacting with corresponding RNA-binding proteins^10–12^. Therefore, identification of potential lncRNA–protein Interactions (LPIs) is vital to understand lncRNAs’ biological functions and mechanisms.

To find new LPIs, many experimental methods were designed^13,14^. However, wet experiments for finding possible LPIs are costly and time-consuming. Computational methods are thus developed as a silver-bullet solution to LPI prediction. This type of methods is classified into two main categories: network-based methods and machine learning-based methods^15,16^.

Network-based LPI prediction methods, for example, random walk with restart-based model^17^, linear neighborhood propagation algorithm^18^, bipartite network projection-based recommendation method^19–21^, HeteSim algorithm^22^, firstly computed lncRNA similarity and protein similarity based on related biological data, and then integrated similarity matrix to heterogeneous lncRNA–protein network, finally designed network propagation algorithms to score for unknown lncRNA–protein pairs. Network-based LPI prediction methods successfully found part of LPIs, however, the type of methods cannot be applied to predict linkage information for an orphan lncRNA or protein.

Machine learning-based LPI identification methods first extracted features of lncRNAs and proteins and then designed a novel machine learning model to compute interaction probabilities for lncRNA–protein pairs. Classical machine learning-based LPI prediction models include matrix factorization-based methods and ensemble learning-based methods. Matrix factorization-based methods represented LPI prediction as a recommender task and used diverse matrix factorization models to discover unobserved LPIs, for example, gradient boosted regression trees^23^, graph regularized nonnegative matrix factorization^24^, and neighborhood regularized logistic matrix factorization^25,26^. Ensemble learning-based methods utilized ensemble techniques and constructed ensemble models for new LPIs identification^27,28^, for example, random forest-based ensemble framework^29^, sequence feature projection-based ensemble algorithm^30^, broad learning system-based stacked ensemble classifier^31^, and graph attention-based deep learning model^32^.

Although computational methods effectively identified potential linkages between lncRNAs and proteins, most of the above models remain the following limitations. First, the performance of these models was evaluated only on one dataset, thereby producing prediction bias. Second, the vast majority of models are not applied to find possible association proteins (or lncRNAs) for lncRNAs (or proteins) without any interaction information. Third, the performance needs to be further improved. To solve the above three problems, in this study, known LPI data are firstly integrated and five different LPI datasets are collected. Second, the features of lncRNAs and proteins are extracted based on four-nucleotide composition and the BioSeq2vec methods, respectively. Finally, a Deep Forest model (LPIDF) with cascade forest structure is designed to find LPI candidates. We compare the proposed LPIDF method with four classical LPI prediction models based on three different cross validations. The results show that LPIDF obtains better average AUCs and the best average AUPRs on the five datasets under the three cross validations. More importantly, case studies demonstrate that most of our predicted lncRNA–protein pairs with higher interaction probabilities are true LPIs and the remaining needs further experimental validation.

## Results

We perform a series of experiments to investigate the prediction performance of our proposed LPIDF method.

### Evaluation metrics

In this study, precision, recall, accuracy, F1-score, AUC and AUPR are used to evaluate the performance of LPIDF. Precision, recall, accuracy, and F1-score are defined as follows.

1 $$Precision=\frac{TP}{TP+FP}$$

2 $$\begin{array}{c} Recall=\frac{TP}{TP+FN} \end{array}$$

3 $$Accuracy=\frac{TP+TN}{TP+FP+TN+FN}$$

4 $$\begin{array}{c}F1-score=\frac{2\times Precision\times Recall}{Precision+Recall} \end{array}$$

where TP, FP, TN, and FN denote the predicted number of true LPIs, false LPIs, true non-LPIs, and false non-LPIs. AUC and AUPR denote the average areas under the ROC curve and the precision-recall curve, respectively. The experiments are repeated for 20 times and the average performance from the 20 rounds is computed as the final performance.

### Experimental settings

In the study, we conduct three different experimental settings.

Five-fold Cross Validation 1 (CV1): Cross validation on lncRNAs, that is, random rows (i.e., lncRNAs) in an LPI matrix $Y$ are masked for testing.

Five-fold Cross Validation 2 (CV2): Cross validation on proteins, that is, random columns (i.e., proteins) in an LPI matrix $Y$ are masked for testing.

Five-fold Cross validation 3 (CV3): Cross validation on lncRNA–protein pairs, that is, random lncRNA–protein pairs in an LPI matrix $Y$ are masked for testing.

Under CV1, in each round, 80% of lncRNAs in an LPI network $Y$ are screened as training set and the remaining is represented as testing set. Under CV2, in each round, 80% of proteins in $Y$ are screened as training set and the remaining is represented as testing set. Under CV3, in each round, 80% of lncRNA–protein pairs in $Y$ are represented as training set and the remaining is represented as testing set. The three cross validations refer to LPI identification for (1) new (unknown) lncRNAs (lncRNAs whose interaction information is unknown), (2) new proteins, and (3) lncRNA–protein pairs, respectively.

### Comparison with four state-of-the-art methods

We compare our proposed LPIDF method with four state-of-the-art association identification methods to evaluate the prediction ability and robustness of LPIDF, that is, XGBoost^33,34^, Categorical Boosting (CatBoost)^35^, random forest^36,37^, and DRPLPI^38^. The above methods are classical machine learning models and obtained wide applications in various areas. XGBoost^33,34^ is a scalable and end-to-end tree boosting-based model. CatBoost^35^ is a novel gradient boosting-based technique and can effectively integrate ordered boosting and processing categorical features. Random forest^36,37^ is composed of multiple decision trees and each tree is independently trained on a random subset. DRPLPI^38^ exploited a multi-head self-attention model to extract high quality LPI features based on long short-term memory encoder-decoder mechanism. In the experiments, we randomly select the same number of negative LPIs as positive LPIs from unknown lncRNA–protein pairs to decrease the overfitting problem produced by data imbalance.

In random forest, the number of trees is set as 70, and the minimum number used to split samples is set as 5. In CatBoost, the maximum number of trees is set as 150, the maximum depth as 15, and the learning rate as 0.5. Other parameters are set as the corresponding values provided by the corresponding manuscript. XGBoost is conducted based on the scikit-learn package^39^.

Table 1 shows the precision, recall, accuracy, F1-score, AUC and AUPR values computed by LPIDF and other four methods under CV1. As shown in Table1, LPIDF achieves the highest average precision, accuracy, F1-score, and AUPR over all datasets, remarkably outperformed other four competing LPI prediction methods. Although the average recall and AUC computed by LPIDF are slightly lower than random forest and DRPLPI, LPIDF obtains the best average AUPR. The computed average AUPR obtained by LPIDF is 0.9022, which is 0.96%, 2.10%, 0.02% and 0.63% higher than XGBoost, CatBoost, random forest, and DRPLPI, respectively. Compared to AUC, AUPR is one more important measurement metric. Therefore, LPIDF can effectively find potential proteins interacting with a new lncRNA.

**Table 1:** The performance of five LPI prediction methods on CV1.

		XGBoost	CatBoost	Random forest	DRPLPI	LPIDF
Precision	Dataset 1	0.8585 ± 0.0199	0.8424 ± 0.0120	0.8357 ± 0.0067	0.8361 ± 0.0086	**0.8621 ± 0.0208**
	Dataset 2	0.8608 ± 0.0120	0.8677 ± 0.0171	0.8529 ± 0.0157	0.8518 ± 0.0167	**0.8716 ± 0.0086**
	Dataset 3	0.7126 ± 0.0210	0.7158 ± 0.0225	0.7236 ± 0.0170	0.7174 ± 0.0195	**0.7285 ± 0.0102**
	Dataset 4	0.8879 ± 0.0495	0.9066 ± 0.0385	0.9248 ± 0.0518	0.9286 ± 0.0335	**0.9374 ± 0.0353**
	Dataset 5	0.8826 ± 0.0124	0.8662 ± 0.0125	0.8882 ± 0.0027	0.8732 ± 0.0133	**0.9000 ± 0.0073**
	Ave	0.8405	0.8397	0.8450	0.8414	**0.8599**
Recall	Dataset 1	0.9179 ± 0.0167	0.9245 ± 0.0041	**0.9593 ± 0.0130**	0.9505 ± 0.0098	0.9170 ± 0.0124
	Dataset 2	0.9289 ± 0.0281	0.9298 ± 0.0159	**0.9740 ± 0.0123**	0.9533 ± 0.0248	0.9183 ± 0.0174
	Dataset 3	0.6979 ± 0.0191	**0.7398 ± 0.0205**	0.7278 ± 0.0083	0.7166 ± 0.0267	0.7199 ± 0.0249
	Dataset 4	**0.6891 ± 0.0571**	0.6879 ± 0.0577	0.6748 ± 0.0408	0.6888 ± 0.0623	0.6722 ± 0.0487
	Dataset 5	0.8531 ± 0.0169	0.8502 ± 0.0110	0.8484 ± 0.0091	**0.8531 ± 0.0124**	0.8476 ± 0.0170
	Ave	0.8174	0.8264	**0.8369**	0.8325	0.8150
Accuracy	Dataset 1	**0.8890 ± 0.0127**	0.8756 ± 0.0067	0.8852 ± 0.0102	0.8821 ± 0.0086	0.8850 ± 0.0090
	Dataset 2	0.8481 ± 0.0109	0.8938 ± 0.0083	**0.9029 ± 0.0085**	0.8934 ± 0.0140	0.8916 ± 0.0083
	Dataset 3	0.7079 ± 0.0095	0.7225 ± 0.0028	0.7226 ± 0.0092	0.7169 ± 0.0104	**0.7254 ± 0.0146**
	Dataset 4	0.8033 ± 0.0383	0.8089 ± 0.0537	0.8049 ± 0.0253	**0.8183 ± 0.0530**	0.8132 ± 0.0284
	Dataset 5	0.8697 ± 0.0098	0.8594 ± 0.0057	0.8708 ± 0.0041	0.8646 ± 0.0068	**0.8767 ± 0.0072**
	Ave	0.8236	0.8320	0.8373	0.8351	**0.8384**
F1-score	Dataset 1	0.8870 ± 0.0111	0.8814 ± 0.0051	**0.8932 ± 0.0080**	0.8876 ± 0.0080	0.8885 ± 0.0091
	Dataset 2	0.8932 ± 0.0118	0.8974 ± 0.0082	**0.9093 ± 0.0084**	0.8993 ± 0.0125	0.8943 ± 0.0088
	Dataset 3	0.7047 ± 0.0094	**0.7270 ± 0.0021**	0.7256 ± 0.0110	0.7165 ± 0.0138	0.7238 ± 0.0085
	Dataset 4	0.7730 ± 0.0290	0.7798 ± 0.0343	0.7702 ± 0.0193	**0.7884 ± 0.0376**	0.7807 ± 0.0186
	Dataset 5	0.8674 ± 0.0071	0.8580 ± 0.0036	0.8608 ± 0.0052	0.8629 ± 0.0036	**0.8729 ± 0.0064**
	Ave	0.8251	0.8287	0.8318	0.8309	**0.8320**
AUC	Dataset 1	0.9387 ± 0.0095	0.9294 ± 0.0057	0.9377 ± 0.0065	0.9333 ± 0.0056	**0.9426 ± 0.0088**
	Dataset 2	0.9403 ± 0.0075	0.9458 ± 0.0070	0.9476 ± 0.0072	0.9408 ± 0.0064	**0.9506 ± 0.0063**
	Dataset 3	0.7975 ± 0.0088	**0.8169 ± 0.0075**	0.8045 ± 0.0153	0.8096 ± 0.0088	0.8108 ± 0.0131
	Dataset 4	0.8677 ± 0.0271	0.8110 ± 0.0291	0.8776 ± 0.0193	**0.8857 ± 0.0251**	0.8480 ± 0.0340
	Dataset 5	0.9518 ± 0.0060	0.8597 ± 0.0054	0.9397 ± 0.0090	0.9472 ± 0.0041	**0.9542 ± 0.0045**
	Ave	0.8992	0.8726	0.9014	**0.9033**	0.9012
AUPR	Dataset 1	0.9196 ± 0.0079	0.9061 ± 0.0052	0.9212 ± 0.0066	0.9106 ± 0.0100	**0.9250 ± 0.0144**
	Dataset 2	0.9214 ± 0.0053	0.9280 ± 0.0087	0.9336 ± 0.0081	0.9222 ± 0.0052	**0.9375 ± 0.0134**
	Dataset 3	0.7663 ± 0.0133	**0.8005 ± 0.0099**	0.7949 ± 0.0162	0.7839 ± 0.0154	0.7964 ± 0.0029
	Dataset 4	0.8995 ± 0.0222	0.8759 ± 0.0260	0.9063 ± 0.0327	**0.9116 ± 0.0179**	0.8937 ± 0.0131
	Dataset 5	0.9564 ± 0.0033	0.8957 ± 0.0056	0.9539 ± 0.0030	0.9510 ± 0.0031	**0.9584 ± 0.0040**
	Ave	0.8926	0.8812	0.9020	0.8959	**0.9022**

The best performance is represented in boldface in each row in each table.

Table 2 gives the comparison results under CV2. In particular, LPIDF computes the best average precision, recall, accuracy, F1-score, AUC and AUPR over all datasets. Over all datasets, LPIDF investigates the best average AUC value of 0.6937, which is 4.80%, 10.81%, 1.17% and 0.91% better than XGBoost, CatBoost, random forest, and DRPLPI, respectively. More importantly, LPIDF calculates the highest average AUPR value of 0.6860, which is 2.17% and 2.65% higher than the second-best and third-best methods, respectively. In summary, under CV2, LPIDF remarkably improves LPI prediction performance compared to the other four prediction methods and is statistically significant in identifying possible lncRNAs for a new protein.

**Table 2:** The performance of five LPI prediction methods on CV2.

		XGBoost	CatBoost	Random forest	DRPLPI	LPIDF
Precision	Dataset 1	0.5630 ± 0.2187	0.2339 ± 0.1389	0.3181 ± 0.2432	0.3426 ± 0.2355	**0.5673 ± 0.2705**
	Dataset 2	0.5214 ± 0.1701	0.4117 ± 0.2269	0.6310 ± 0.1672	**0.6634 ± 0.2152**	0.6374 ± 0.1278
	Dataset 3	0.6444 ± 0.0759	0.5885 ± 0.1198	0.6873 ± 0.2617	**0.7173 ± 0.0554**	0.6248 ± 0.1310
	Dataset 4	0.4502 ± 0.1057	0.5185 ± 0.1633	**0.5597 ± 0.2284**	0.4951 ± 0.1616	0.5100 ± 0.0385
	Dataset 5	0.6798 ± 0.1338	0.7454 ± 0.1015	0.7516 ± 0.0375	**0.7562 ± 0.1097**	0.6976 ± 0.0768
	Ave	0.5718	0.4996	0.5895	0.5949	**0.6074**
Recall	Dataset 1	**0.1205 ± 0.0735**	0.0898 ± 0.0569	0.0056 ± 0.0086	0.0056 ± 0.0041	0.0996 ± 0.1279
	Dataset 2	0.0458 ± 0.0278	0.1162 ± 0.1111	0.0136 ± 0.0087	0.0159 ± 0.0094	**0.1418 ± 0.1116**
	Dataset 3	0.3651 ± 0.1738	0.5795 ± 0.1973	0.2578 ± 0.1301	0.3695 ± 0.1541	**0.6318 ± 0.2191**
	Dataset 4	0.9087 ± 0.0993	0.7777 ± 0.1343	**0.9899 ± 0.0123**	0.8619 ± 0.1062	0.9284 ± 0.0398
	Dataset 5	0.9654 ± 0.0244	0.9096 ± 0.0543	0.9545 ± 0.0287	0.9219 ± 0.0516	**0.9762 ± 0.0189**
	Ave	0.4811	0.4946	0.4443	0.4350	**0.5556**
Accuracy	Dataset 1	0.5499 ± 0.1385	0.4727 ± 0.1757	0.5398 ± 0.1417	0.5383 ± 0.1533	**0.5631 ± 0.1793**
	Dataset 2	0.5386 ± 0.1497	0.5237 ± 0.1041	0.5125 ± 0.0845	0.5422 ± 0.1518	**0.5596 ± 0.1162**
	Dataset 3	0.5822 ± 0.0747	0.5972 ± 0.1037	0.5901 ± 0.1071	0.6159 ± 0.0809	**0.6187 ± 0.0812**
	Dataset 4	0.4516 ± 0.1335	0.5286 ± 0.1404	**0.5571 ± 0.2191**	0.4972 ± 0.1564	0.5147 ± 0.0313
	Dataset 5	0.7353 ± 0.1020	0.7909 ± 0.0546	**0.8197 ± 0.0311**	0.8029 ± 0.0608	0.7736 ± 0.0546
	Ave	0.5715	0.5826	0.6038	0.5993	**0.6059**
F1-score	Dataset 1	0.1803 ± 0.1002	0.1181 ± 0.0905	0.0109 ± 0.0166	0.0107 ± 0.0076	0.1461 ± 0.1693
	Dataset 2	0.0819 ± 0.0468	0.1680 ± 0.1460	0.0261 ± 0.0160	0.0308 ± 0.0179	**0.2146 ± 0.1560**
	Dataset 3	0.4425 ± 0.1306	0.5465 ± 0.1386	0.3349 ± 0.1578	0.4708 ± 0.1367	**0.5954 ± 0.1125**
	Dataset 4	0.5954 ± 0.0962	0.5970 ± 0.0707	**0.6901 ± 0.1579**	0.6085 ± 0.0890	0.6565 ± 0.0276
	Dataset 5	0.7889 ± 0.0941	0.8146 ± 0.0656	**0.8407 ± 0.0321**	0.8253 ± 0.0691	0.8110 ± 0.0518
	Ave	0.4178	0.4488	0.3805	0.3892	**0.4847**
AUC	Dataset 1	0.6116 ± 0.1384	0.4431 ± 0.0607	0.6034 ± 0.1648	0.5407 ± 0.1431	**0.6549 ± 0.1973**
	Dataset 2	0.5819 ± 0.0788	0.5090 ± 0.0427	**0.6079 ± 0.0490**	0.5938 ± 0.1076	0.5956 ± 0.1482
	Dataset 3	0.6239 ± 0.0781	0.6236 ± 0.0846	0.6402 ± 0.0683	0.6698 ± 0.0811	**0.7224 ± 0.1072**
	Dataset 4	0.5515 ± 0.1363	0.5634 ± 0.1026	0.6414 ± 0.1523	**0.7190 ± 0.0665**	0.5794 ± 0.1465
	Dataset 5	0.8554 ± 0.0936	0.7889 ± 0.0411	**0.9169 ± 0.0401**	0.8998 ± 0.0563	0.9161 ± 0.0397
	Ave	0.6457	0.5856	0.6820	0.6846	**0.6937**
AUPR	Dataset 1	0.5460 ± 0.1510	0.3720 ± 0.1218	0.5629 ± 0.1278	0.4744 ± 0.1726	**0.5937 ± 0.1696**
	Dataset 2	0.5099 ± 0.1366	0.4746 ± 0.1614	0.5409 ± 0.0952	0.5240 ± 0.1519	**0.5611 ± 0.1563**
	Dataset 3	0.6241 ± 0.0709	0.6925 ± 0.0945	0.6061 ± 0.2265	0.6801 ± 0.0722	**0.7041 ± 0.1431**
	Dataset 4	0.5640 ± 0.1383	0.6999 ± 0.0586	0.6900 ± 0.2169	**0.7518 ± 0.0611**	0.6750 ± 0.0770
	Dataset 5	0.8267 ± 0.1761	0.8522 ± 0.0559	**0.8978 ± 0.0564**	0.8912 ± 0.0672	0.8962 ± 0.0614
	Ave	0.6141	0.6182	0.6595	0.6643	**0.6860**

The best performance is represented in boldface in each row in each table.

The prediction results computed under CV3 are shown in Table 3. In particular, LPIDF outperforms other LPI prediction methods over all datasets in terms of all six measurements. For example, LPIDF achieves the best average AUC value of 0.9457, which is 1.72%, 6.39%, 0.87%, and 0.97% better than XGBoost, CatBoost, random forest, and DRPLPI, respectively. In addition, for the AUPR metric, LPIDF obtains the best average AUPR of 0.9382, which is 0.88% and 1.20% superior to the second-best and third-best methods, respectively. It can be seen that the LPIDF can effectively predict potential LPIs.

**Table 3:** The performance of five LPI prediction methods on CV3.

		XGBoost	CatBoost	Random forest	DRPLPI	LPIDF
Precision	Dataset 1	0.8508 ± 0.0115	0.8457 ± 0.0142	0.8466 ± 0.0056	0.8401 ± 0.0131	**0.8589 ± 0.0115**
	Dataset 2	**0.8682 ± 0.0102**	0.8604 ± 0.0121	0.8549 ± 0.0065	0.8574 ± 0.0112	0.8645 ± 0.0119
	Dataset 3	0.7455 ± 0.0213	0.7401 ± 0.0156	0.7438 ± 0.0171	0.7503 ± 0.0198	**0.7549 ± 0.0120**
	Dataset 4	0.9117 ± 0.0051	0.9340 ± 0.0134	0.9261 ± 0.0171	0.9381 ± 0.0142	**0.9390 ± 0.0227**
	Dataset 5	0.8899 ± 0.0057	0.9250 ± 0.0047	0.9223 ± 0.0017	0.9224 ± 0.0040	**0.9297 ± 0.0039**
	Ave	0.8532	0.8610	0.8587	0.8617	**0.8694**
Recall	Dataset 1	0.9293 ± 0.0079	0.9676 ± 0.0065	**0.9707 ± 0.0016**	0.9630 ± 0.0092	0.9634 ± 0.0104
	Dataset 2	0.9486 ± 0.0083	0.9666 ± 0.0088	0.9745 ± 0.0041	0.9722 ± 0.0048	**0.9752 ± 0.0079**
	Dataset 3	0.7863 ± 0.0157	0.8031 ± 0.0240	0.8061 ± 0.0134	0.7976 ± 0.0128	**0.8257 ± 0.0149**
	Dataset 4	0.8394 ± 0.0305	0.8734 ± 0.0479	0.8803 ± 0.0235	0.8711 ± 0.0479	**0.8908 ± 0.0225**
	Dataset 5	0.9048 ± 0.0051	0.9304 ± 0.0064	0.9282 ± 0.0034	**0.9345 ± 0.0047**	0.9307 ± 0.0047
	Ave	0.8817	0.9082	0.9120	0.9077	**0.9172**
Accuracy	Dataset 1	0.8832 ± 0.0063	0.8957 ± 0.0083	0.8974 ± 0.0051	0.8899 ± 0.0080	**0.9025 ± 0.0065**
	Dataset 2	0.9022 ± 0.0072	0.9049 ± 0.0090	0.9046 ± 0.0039	0.9051 ± 0.0064	**0.9111 ± 0.0075**
	Dataset 3	0.7591 ± 0.0151	0.7608 ± 0.0129	0.7640 ± 0.0114	0.7660 ± 0.0136	**0.7786 ± 0.0112**
	Dataset 4	0.8792 ± 0.0143	0.9056 ± 0.0234	0.9056 ± 0.0176	0.9066 ± 0.0214	**0.9161 ± 0.0066**
	Dataset 5	0.8964 ± 0.0027	0.9279 ± 0.0049	0.9250 ± 0.0023	0.9283 ± 0.0037	**0.9302 ± 0.0027**
	Ave	0.8640	0.8790	0.8793	0.8792	**0.8877**
F1-score	Dataset 1	0.8883 ± 0.0070	0.9025 ± 0.0091	0.9044 ± 0.0038	0.8973 ± 0.0085	**0.9080 ± 0.0062**
	Dataset 2	0.9066 ± 0.0062	0.9103 ± 0.0094	0.9108 ± 0.0042	0.9111 ± 0.0054	**0.9164 ± 0.0074**
	Dataset 3	0.7653 ± 0.0173	0.7702 ± 0.0170	0.7735 ± 0.0104	0.7731 ± 0.0212	**0.7886 ± 0.0096**
	Dataset 4	0.8738 ± 0.0173	0.9019 ± 0.0260	0.9025 ± 0.0197	0.9025 ± 0.0248	**0.9138 ± 0.0071**
	Dataset 5	0.8973 ± 0.0047	0.9276 ± 0.0049	0.9252 ± 0.0018	0.9284 ± 0.0033	**0.9302 ± 0.0030**
	Ave	0.8663	0.8825	0.8833	0.8825	**0.8914**
AUC	Dataset 1	0.9376 ± 0.0054	0.8955 ± 0.0046	0.9484 ± 0.0031	0.9413 ± 0.0038	**0.9521 ± 0.0053**
	Dataset 2	0.9507 ± 0.0040	0.9049 ± 0.0083	0.9537 ± 0.0051	0.9510 ± 0.0064	**0.9545 ± 0.0064**
	Dataset 3	0.8452 ± 0.0133	0.7755 ± 0.0099	0.8531 ± 0.0096	0.8517 ± 0.0106	**0.8739 ± 0.0111**
	Dataset 4	0.9407 ± 0.0098	0.9054 ± 0.0228	0.9483 ± 0.0164	0.9526 ± 0.0169	**0.9634 ± 0.0091**
	Dataset 5	0.9681 ± 0.0008	0.9279 ± 0.0050	0.9815 ± 0.0006	0.9834 ± 0.0005	**0.9848 ± 0.0008**
	Ave	0.9285	0.8818	0.9370	0.9360	**0.9457**
AUPR	Dataset 1	0.9155 ± 0.0074	0.9259 ± 0.0070	0.9279 ± 0.0101	0.9212 ± 0.0085	**0.9381 ± 0.0095**
	Dataset 2	0.9314 ± 0.0086	0.9218 ± 0.0075	**0.9403 ± 0.0088**	0.9341 ± 0.0113	0.9384 ± 0.0101
	Dataset 3	0.8213 ± 0.0204	0.8235 ± 0.0153	0.8350 ± 0.0110	0.8271 ± 0.0186	**0.8562 ± 0.0135**
	Dataset 4	0.9532 ± 0.0083	0.9354 ± 0.0127	0.9615 ± 0.0129	0.9648 ± 0.0122	**0.9726 ± 0.0043**
	Dataset 5	0.9709 ± 0.0012	0.9450 ± 0.0037	0.9824 ± 0.0005	0.9839 ± 0.0005	**0.9857 ± 0.0008**
	Ave	0.9185	0.9103	0.9294	0.9262	**0.9382**

The best performance is represented in boldface in each row in each table.

### Case study

After confirming the performance of our proposed LPIDF method, we further identify possible LPIs, especially predict interaction information for new lncRNAs and proteins.

#### Finding possible proteins interacting with new lncRNAs

In this section, we intend to find potential proteins interacting with new lncRNAs. Small Nucleolar RNA Host Gene 3 (SNHG3) and Growth Arrest-Special transcript 5 (GAS5) are masked all association information and taken as new lncRNAs. LPIDF is then applied to identify possible proteins interacting with the two lncRNAs.

SNHG3 is an RNA Gene affiliated with the lncRNA class. It may have dense correlation with various cancers, for example, hepatocellular carcinoma^40^, non-small-cell lung cancer^41^, clear cell renal cell carcinoma^42^, gastric cancer^43^, hypoxic-ischemic brain damage^44^, papillary thyroid carcinoma^45^, ovarian cancer^46,47^, bladder cancer^48^, and acute myeloid leukemia^49^. Table 4 shows the predicted top 5 proteins related to SNHG3 with the highest interaction probabilities on three human datasets.

**Table 4:** The predicted top 5 proteins interacting with SNHG3.

Dataset	Proteins	Confirmed	LPIDF	XGBoost	Random forest	CatBoost	DRPLPI
Dataset1	Q15717	Yes	1	1	2	1	2
	P35637	Yes	2	5	4	7	3
	O00425	Yes	3	2	6	5	1
	Q9UKV8	Yes	4	6	1	8	6
	Q9NZI8	Yes	5	3	8	3	5
Dataset2	Q15717	Yes	1	1	2	1	1
	Q9NZI8	Yes	2	3	7	4	8
	Q9Y6M1	Yes	3	2	5	3	2
	P35637	Yes	4	4	1	5	4
	Q96PU8	Yes	5	18	16	15	17
Dataset3	Q9NUL5	No	1	1	1	1	1
	Q9Y6M1	Yes	2	3	2	20	4
	Q9NZI8	Yes	3	4	5	7	2
	O00425	No	4	2	3	3	3
	Q13148	No	5	7	8	5	4

The results from Table 4 show that SNHG3-protein interaction pairs predicted by LPIDF are rank advanced in all other four methods. We predict that O00425 may interact with SNHG3 (ranked as 4) in dataset 3, which has been validated in dataset 1. In addition, we observe that Q9NUL5 and Q13148 may interact with SNHG3. Among all possible 27 proteins, the interaction between Q9NUL5 and SNHG3 is ranked as 1 by all five LPI prediction methods. The association between Q13148 and SNHG3 is ranked as 5, 7, 8, 5, and 4 by LPIDF, XGBoost, random forest, CatBoost, and DRPLIP, respectively. The facts demonstrate the powerful prediction performance of LPIDF.

GAS5 can prevent glucocorticoid receptors from being activated and thus control transcriptional activities from its target genes. It is inferred as a potential tumor suppressor and has close correlations with coronary artery disease^50^, cirrhotic livers^51^, coronary artery disease^52,53^, rheumatoid arthritis^54^, Parkinson’s disease^55^, and primary glioblastoma^56^.

Table 5 lists the predicted top 5 proteins interacting with GAS3 with the highest association scores on three human datasets. In dataset 3, although the interactions between GAS5 and Q9NZI8 and Q9Y6M1 are unknown, we find that the two LPIs are ranked as 5 and 4 by LPIDF, respectively. More importantly, in datasets 1 and 2, it can be seen that Q9NZI8 and Q9Y6M1 show higher interaction probabilities with GAS5 and the two LPIs have been reported. In addition, O00425 is inferred to interact with GAS5 with the ranking of 2 in dataset 3 and has been validated in dataset 1. These facts again suggest that LPIDF can effectively find possible proteins associated with a new lncRNA.

**Table 5:** The predicted top 5 proteins interacting with GAS5.

Dataset	Proteins	Confirmed	LPIDF	XGBoost	Random forest	CatBoost	DRPLPI
Dataset1	O00425	Yes	1	2	1	6	1
	Q15717	No	2	1	2	1	2
	P35637	No	3	4	3	5	3
	Q9NZI8	Yes	4	3	4	2	5
	Q9Y6M1	Yes	5	5	5	3	4
Dataset2	Q15717	No	1	1	3	8	1
	Q9NZI8	Yes	2	3	6	2	5
	Q9Y6M1	Yes	3	2	4	5	3
	P35637	No	4	4	2	1	2
	P31483	Yes	5	5	1	3	4
Dataset3	Q9NUL5	Yes	1	1	1	1	1
	O00425	No	2	2	2	3	10
	Q07955	Yes	3	9	6	12	2
	Q9Y6M1	No	4	3	3	5	6
	Q9NZI8	No	5	4	4	2	5

#### Finding potential lncRNAs interacting with new proteins

We continue to uncover lncRNAs interacting with a new protein on three human datasets. Q13148 and Q9HCK5 are masked all associated lncRNAs and taken as new proteins. LPIDF is then used to find possible associated lncRNAs for the two proteins.

Q13148 is an RNA-binding protein involved in RNA biogenesis and processing and various neurodegenerative diseases^57–60^. In addition, it also participates in the formation and regeneration of normal skeletal muscles and plays an important role in keeping the circadian clock periodicity^59,60^. Its second RNA recognition motif has been reported as a major promoter towards aggregation and resultant toxicity^61^. Frontotemporal lobar degeneration associated with Q13148 aggregation is depicted as progressive neuronal atrophy in cerebral cortex^62^. Table 6 illustrates the predicted top 5 lncRNAs associated with Q13148 on three human datasets. From Table 6, we can investigate that all predicted top 5 lncRNAs interacting with Q13148 are known in the three datasets.

**Table 6:** The predicted top 5 lncRNAs interacting with Q13148.

Dataset	lncRNAs	Confirmed	LPIDF	XGBoost	CatBoost	Random forest	DRPLPI
Dataset1	SNHG1	Yes	1	53	60	17	13
	NEAT1	Yes	2	113	52	30	199
	7SL	Yes	3	784	234	472	264
	RP11-439E19.10	Yes	4	376	14	415	55
	SFPQ	Yes	5	28	25	18	7
Dataset2	SNHG1	Yes	1	14	22	7	5
	NEAT1	Yes	2	5	128	1	39
	7SL	Yes	3	61	14	4	150
	RP11-439E19.10	Yes	4	274	103	106	559
	SFPQ	Yes	5	48	13	8	66
Dataset3	RPI001_124073	Yes	1	4	1	6	59
	LINC00638	Yes	2	1	707	2	2
	LINC00338	Yes	3	28	637	4	7
	RP11-38P22.2	Yes	4	29	461	99	47
	GAS5	Yes	5	110	8	13	110

Table 7 lists the identified top 5 lncRNAs associated with Q9HCK5 on three human datasets. Q9HCK5 is required for RNA-mediated genes’ silencing, RNA-directed transcription and human hepatitis delta virus replication^63^. Table 7 demonstrates that all predicted top 5 LPIs for Q9NCK5 are given in the three datasets. In summary, LPIDF may be appropriate for LPI identification for a new protein.

**Table 7:** The predicted top 5 lncRNAs interacting with Q9HCK5.

Dataset	lncRNAs	Confirmed	LPIDF	XGBoost	CatBoost	Random forest	DRPLPI
Dataset1	RPI001_233996	Yes	1	468	73	31	71
	RPI001_122583	Yes	2	16	41	139	35
	RPI001_1006381	Yes	3	44	23	38	177
	RPI001_1000866	Yes	4	580	51	15	189
	RP5-1057J7.6	Yes	5	263	29	56	116
Dataset2	SFPQ	Yes	1	45	1	2	23
	RPI001_1015379	Yes	2	55	91	75	2
	RPI001_247329	Yes	3	126	51	22	24
	RPI001_1000866	Yes	4	18	5	36	4
	NEAT1	Yes	5	15	49	4	9
Dataset3	RP11-357C3.3	Yes	1	7	6	41	36
	RP1-140A9.1	Yes	2	6	390	15	11
	RPI001_124073	Yes	3	5	25	9	32
	RPI001_1001088	Yes	4	1	14	10	10
	AC010890.1	Yes	5	17	42	62	8

#### Finding new LPIs based on known LPIs

**Figure 1:**
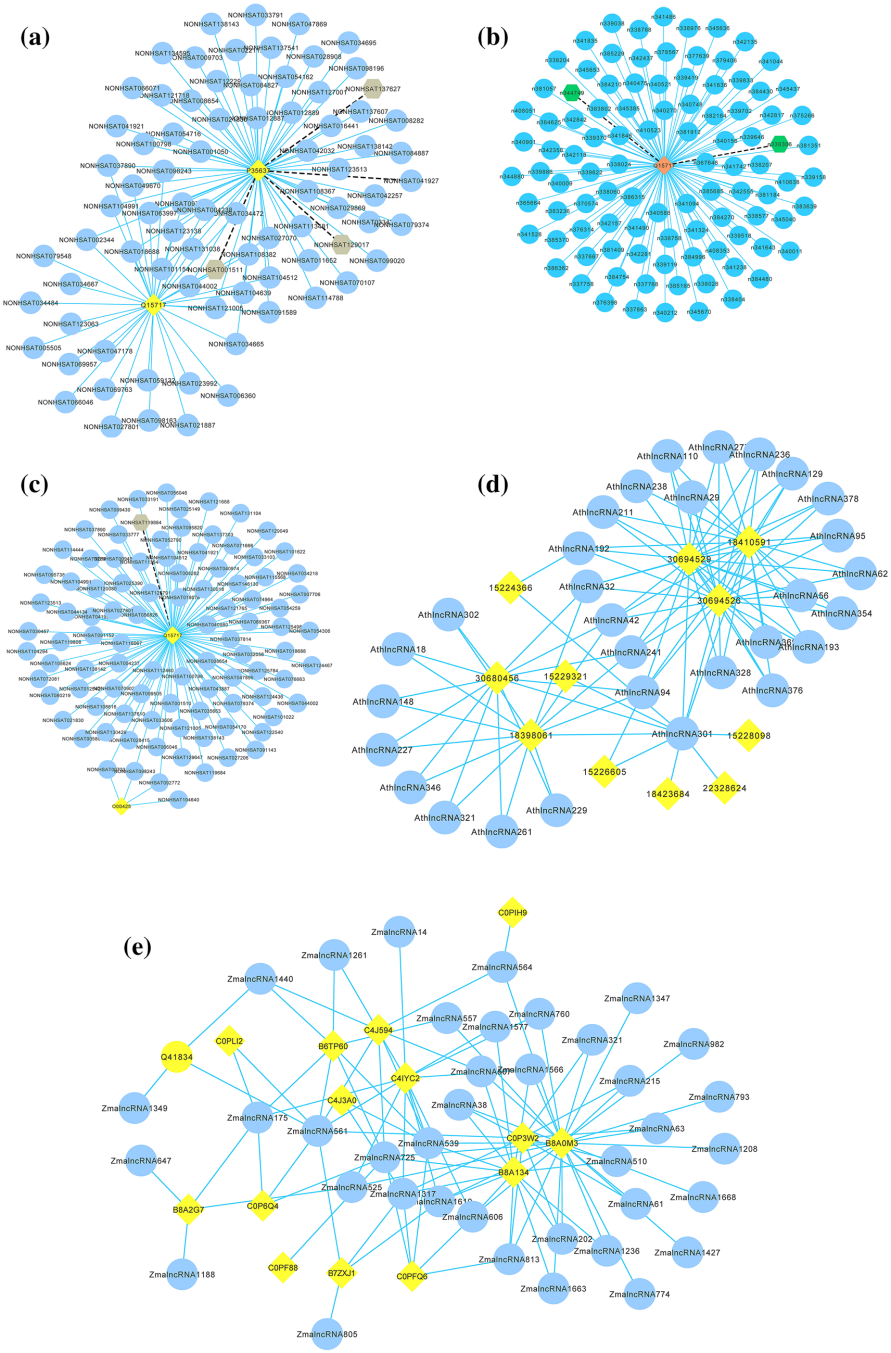
The predicted top 100 LPIs on the five datasets **(a)** Dataset 1, **(b)** Dataset 2, **(c)** Dataset 3, **(d)** Dataset 4, **(e)** Dataset 5.

The number of lncRNA–protein pairs with unknown interaction information is 51,686, 71,075, 22,572, 2,867 and 49,435 on the five datasets, respectively. We rank these unknown lncRNA–protein pairs based on their interaction probabilities computed by LPIDF and list the predicted top 100 lncRNA–protein pairs. The results are shown in Fig. 1. In Fig. 1, black dotted lines and sky blue solid lines represent unknown and known LPIs predicted by LPIDF, respectively. Tan hexagons and light sky blue circulars denote lncRNAs whose interactions with proteins are unknown and known, respectively. Yellow diamonds denote proteins.

We observe that some identified lncRNA–protein pairs have higher interaction probabilities. For example, the interactions between NONHSAT137627 and P35637, n344749 and Q15717, NONHSAT119864 and Q15717, AthIncRNA18 and Q9LES2, and ZmaLncRNA38 and C4J594 are ranked as 33, 97, 85, 161, and 215, respectively. The lncRNA–protein pairs with advanced ranks need further experimental validation.

The lncRNA FTX (NONHSAT137627) can positively regulate the expression and function of ALG3 in AML cells, especially cell growth and apoptosis related to ADR-resistance. FTX could thus probably be applied to reduce therapeutic resistance in AML^64^. P35637 plays a key role in RNA transport, mRNA stability and synaptic homeostasis in neuronal cells^63^. The protein has been validated to be target of the treatment of cancers, amyotrophic lateral sclerosis, and Alzheimer’s disease^65^.

In dataset 2, it is observed that FTX interacts with Q15717, Q9NZI8, and P26599. Q15717 helps in increasing the leptin mRNA’s stability. Q9NZI8 can regulate neurite outgrowth and neuronal cell migration, promote tumor-derived cells’ adhesion and movement, and prevent infectious HIV-1 particles’ formation^64^. P26599 can bind to the viral internal ribosome entry site and stimulate the translation mediated by the picornaviruses’ infection site. Q35637 has similar functions with Q15717, Q9NZI8, and P26599. Based on the “guilt-by-association” theory, we infer that FTX may associate with P35637.

#### Fractions of true LPIs among the predicted top N LPIs

In addition, we consider the fractions of true LPIs among the inferred top $N$ LPIs. The results are shown in Table 8. $N$ is selected as 10, 30, and 50, respectively. From Table 8, we can find that all the predicted top 10 LPIs by LPIDF have been labeled as 1 on five datasets. Similar to top 10, we can obtain the same fraction results on the predicted top 30 LPIs. For the predicted top 50 LPIs by LPIDF, although only 94% of LPIs have been labeled as 1 in dataset 1, all the top 50 LPIs are known on other four datasets. In summary, LPIDF obtains the best prediction performance based on fractions of true LPIs among the top 10, 30, and 50 LPIs.

**Table 8:** The fractions of true LPIs among the top $N$ interactions under CV3.

		XGBoost (%)	CatBoost (%)	Random forest (%)	DRPLPI (%)	LPIDF (%)
Dataset 1	Top 10	60	100	90	100	100
	Top 30	70	90	90	100	100
	Top 50	74	88	88	94	94
Dataset 2	Top 10	70	70	90	90	100
	Top 30	77	87	87	93	100
	Top 50	80	86	86	92	100
Dataset 3	Top 10	80	90	90	100	100
	Top 30	93	93	96	50	100
	Top 50	88	96	98	96	100
Dataset 4	Top 10	100	100	100	100	100
	Top 30	97	100	100	100	100
	Top 50	96	100	94	100	100
Dataset 5	Top 10	100	100	90	100	100
	Top 30	100	100	86	100	100
	Top 50	100	100	88	100	100

## Discussion and conclusion

lncRNAs are widely distributed in various organisms and regulate gene expression on transcriptome and post-transcriptome. However, lncRNAs are difficult to crystallize and only several lncRNAs have been investigated. Since lncRNAs play an important regulatory role in protein molecules, the discovery of proteins binding to specific lncRNAs becomes an issue to identify lncRNAs’ functions and mechanisms.

In this study, first, we integrate five LPI datasets where three datasets are from human and the remaining is from plants. Second, features of lncRNAs and proteins are selected by four-nucleotide composition and BioSeq2vec based on their sequences, respectively. Finally, a deep forest model with cascade forest structure, LPIDF, is developed to predict LPI candidates. To evaluate the performance of LPIDF, we compare our proposed LPIDF method with other four LPI prediction models on five datasets under three cross validations. The results suggest that LPIDF remarkably outperforms other four competing LPI identification methods. We further conduct a series of case studies to find possible associated proteins (or lncRNAs) for new lncRNAs (or proteins) and potential LPIs. The results from case analyses again demonstrate that LPIDF is a powerful LPI identification method.

LPIDF can compute the optimal precision, recall, accuracy, F1-score, AUC and AUPR. We think that it may be attribute to the following advantages. First, LPIDF selects high quality features of lncRNAs and proteins based on four-nucleotide composition and BioSeq2vec, respectively. Second, deep forest with cascade forest structure could automatically determine the depths of cascade forest, thereby reducing prediction bias produced by parameter tuning. Finally, each layer in the cascade forest receives LPI features from the last layer and sends its result to the next layer. Since all layers are automatically generated, LPIDF need not set too many hyperparameters. The predominant experimental consequences indicate that LPIDF has a powerful ability in excavating new LPIs.

In addition, the time required for the proposed LPIDF model and other methods is investigated. The details are shown in Table 9. It can be seen that the time required for LPIDF is much lower than ones of CatBoost and DRPLPI.

**Table 9:** The time required for all LPI prediction methods.

	XGBoost	CatBoost	Random forest	DRPLPI	LPIDF
Dataset 1	168 s	74,120 s	20 s	74,292 s	28,174 s
Dataset 2	688 s	70,042 s	22 s	68,469 s	25,348 s
Dataset 3	527 s	63,627 s	37 s	63,711 s	26,342 s
Dataset 4	400 s	25,971 s	6 s	25,994 s	7216 s
Dataset 5	1852 s	61,998 s	197 s	62,258 s	82,022 s

Where s denotes second.

However, our work has a few limitations. We only consider LPI prediction on human and plant LPI-related datasets. Indeed, other species closer human evolutionarily than plants should be investigated. In addition, the predicted LPIs with the highest interaction probabilities should be experimentally validated.

In the future, first, we will integrate more biological information, for example, disease symptom information, drug chemical structure, miRNA-lncRNA interactions. Second, we will consider the prediction performance of the proposed model on other species closer human evolutionarily than plants. Third, CD-Hit^66^ is one broadly used software for reducing sequence redundancy. To improve the performance of sequence analyses algorithms, we will further remove proteins with high sequence similarity in larger datasets based on CD-Hit. Finally, we will further conduct experimental validation for the predicted RNA-binding proteins.

## Materials and methods

### Data preparation

In this study, we integrate five different LPI datasets. Dataset 1 was provided by Li et al.^17^. Noncoding RNA–protein interaction data were firstly downloaded from the NPInter 2.0 database^67^. lncRNA and protein sequences were extracted from the NONCODE database 4.0^68^ and the UniProt^65^ database, respectively. 3,487 LPIs from 938 lncRNAs and 59 proteins were obtained. We then remove lncRNAs and proteins whose sequences are unknown in the UniProt^65^, NPInter^67^ and NONCODE^68^ databases. Finally, we obtain 3,479 LPIs from 935 lncRNAs and 59 proteins.

Dataset 2 was provided by Zheng et al.^22^. Noncoding RNA–protein interaction, lncRNA and protein sequences were downloaded from NPInter 2.0^67^, NONCODE 4.0^68^, and UniProt^65^, respectively. They obtained 4,467 LPIs between 1,050 lncRNAs and 84 proteins. Similar to dataset 1, we further remove the lncRNAs and proteins whose sequences are unknown in the NONCODE^68^, UniProt^65^, and NPInter^67^ databases and obtain 3,265 LPIs from 885 lncRNAs and 84 proteins.

Dataset 3 was provided by Zhang et al.^18^. Experimentally validated LPIs between 1,114 lncRNAs and 96 proteins were extracted based on data resources compiled by Ge et al.^69^. The sequence and expression data of lncRNAs in 24 human tissues or cell types were downloaded from the NONCODE 4.0 database^68^. The sequence data of proteins were obtained from the SUPERFAMILY database^70^. lncRNAs without sequence or expression information and proteins without sequence information were removed. lncRNA (or protein) with only one associated protein (or lncRNA) were still removed. Finally, 4,158 LPIs from 990 lncRNAs and 27 proteins were selected.

Dataset 4 contains sequence information of lncRNAs and proteins about *Arabidopsis thaliana* from the plant lncRNA database (PlncRNADB^71^). LPI data can be obtained from http://bis.zju.edu.cn/plncRNADB. The dataset contains 948 LPIs from 109 lncRNAs and 35 proteins.

Dataset 5 contains sequence data of lncRNAs and proteins about *Zea mays* from the PlncRNADB database^71^. LPI data can be downloaded from http://bis.zju.edu.cn/plncRNADB. The dataset contains 22,133 LPIs from 1,704 lncRNAs and 42 proteins. Table 10 describes the details about the five datasets.

**Table 10:** The details of LPI data.

Dataset	lncRNAs	Proteins	LPIs
Dataset 1	935	59	3479
Dataset 2	885	84	3265
Dataset 3	990	27	4158
Dataset 4	109	35	948
Dataset 5	1704	42	22,133

We describe an LPI network as a matrix $Y$:

5 $${Y}_{ij}=\left\{\begin{array}{ll}1& \mathrm{if}\hspace{0.17em}\mathrm{ lncRNA }{l}_{i}\hspace{0.25em}\hspace{0.17em}\mathrm{interacts}\hspace{0.25em}\hspace{0.17em}\mathrm{with\,\, protein}\hspace{0.25em}{p}_{j}\\ 0& \mathrm{otherwise}\end{array}\right.$$

### Feature selection

#### Feature selection of lncRNAs

Tri-nucleotide composition is effectively applied to characterize lncRNA sequences^72^. In this section, we use four-nucleotide composition to select lncRNA features. Given an lncRNA sequence $L$ with the length of $x$ where ${l}_{i}\in \{\mathrm{A},\mathrm{C},\mathrm{G},\mathrm{T}\}$ and $i=\mathrm{1,2}, ...,x$, we use a four-tuple letter arrangement, for example, (A, A, A, A), (A, A, A, C), (A, A, A, G), …, (T, T, T, T), to compute the numeric matrix from $L$.

#### Feature selection of proteins

The encoder-decoder structure can better describe sequence-to-sequence features^73,74^. Inspired by the sequence representation techniques provided by Sutskever et al.^74^ and Yi et al.^75^, we use Biological Sequence-to-vector (BioSeq2vec) representation learning method^75^ with encoder-decoder structure to characterize amino acids of a protein.

**Figure 2:**
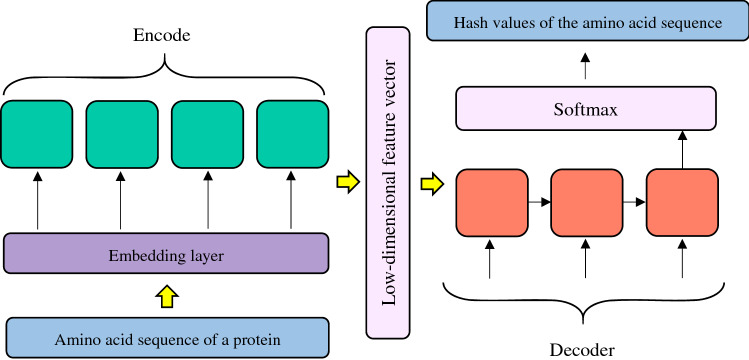
Protein feature selection based on the encoder–decoder.

For a protein with sequence length of $L$, first, a sliding window of size $K$ is used to divide the sequence into $L-K+1$ segments. Second, the segments are converted into hash values. Finally, the hash values are used as input of an autoencoder. As shown in Fig. 2, an input vector composed of the hash values is first mapped into a low-dimensional feature vector by an encoder. Second, the low-dimensional feature vector is reproduced as an input vector by a decoder. Finally, the reproduced low-dimensional feature vector in the final intermediate layer is used as features of a protein.

### Deep forest with cascade forest structure

In this study, we utilize a Deep Forest with cascade forest structure (LPIDF) to find new LPIs. Deep forest with cascade forest structure, integrating deep forest and ensemble learning, exploits an ensemble-ensemble architecture. In the model, deep forest^76^ conducts layer-by-layer propagation and feature transformation. Ensemble learning-based model, composed of multiple single classifiers, more effectively improves LPI prediction compared with one single classifier^77^. For ensemble learning, larger diversities between single classifiers mean better improvement. To ensure the diversity, in this study, four different types of classifiers, logistic regression, XGBoost Classifier, random forest, and extra trees, are utilized to learn the model.

**Figure 3:**
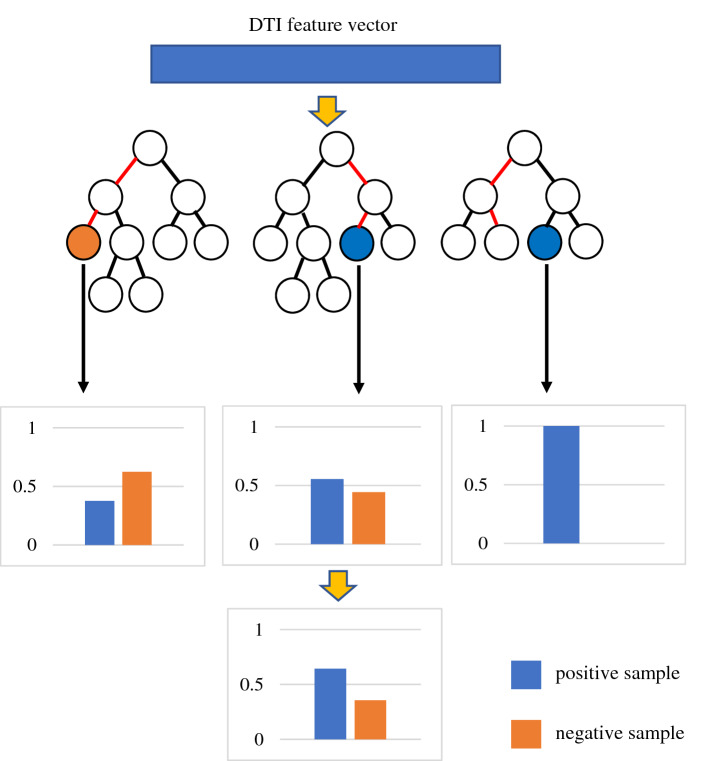
Computing the probability that a feature is classified as positive (or negative) sample.

In the model, class vectors used to denote the class distribution are obtained through the four basic classifiers. For a given LPI feature, the class distribution first calculated the proportions that the feature classifies an lncRNA–protein pair as two classes (positive class and negative class), respectively. Suppose that there are three trees in a random forest. As shown in Fig. 3, for a LPI feature ${f}_{i}$, the probabilities that ${f}_{i}$ classify an lncRNA–protein pair as two classes (positive class and negative class) in the three trees are ${(\mathrm{0.3750,0.6250})}^{T}$, ${(\mathrm{0.5556,0.4444})}^{T}$ and ${(\mathrm{1.0000,0}.0000)}^{T}$, respectively. The probabilities are then summed up and averaged and thus the final class distribution ${(\mathrm{0.6435,0.3565})}^{T}$ can be computed based on the feature ${f}_{i}$. That is, the probability that ${f}_{i}$ classify the lncRNA–protein pair as positive example is $(0.3750+0.5556+1.0000)/3=0.6435$ and the probability that ${f}_{i}$ classify the lncRNA–protein pair as negative sample is $(0.6250+0.4444+0.0000)/3=0.3565$.

Similarly, at each layer, for each LPI feature, logistic regression, XGBoost Classifier, random forest, and extra trees are trained. An 8-dimensional class vector is generated based on two classes and four types of classifiers.

**Figure 4:**
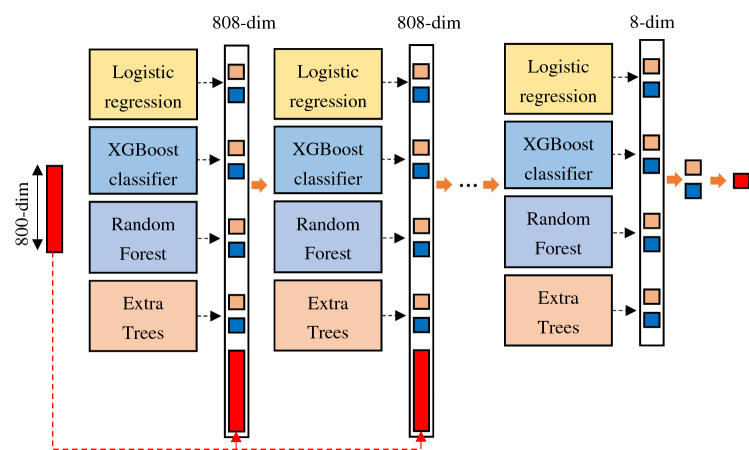
Deep forest with cascade forest structure.

Figure 4 shows a deep forest with cascade structure. As illustrates in Fig. 4, an 800-dimensional feature vector is used as the initial input to the cascade forest. After each layer, the generated eight-dimensional class vector with the most important information combining the old 800-dimensional features are used as the input at the next layer. The details are shown as follows. First, four different types of classifiers, logistic regression, XGBoost Classifier, random forest, and extra trees, are utilized to train the model. Second, an eight-dimensional class vector is picked and concatenated with the original 800-dimensional feature vector to generate an 808-dimensional vector. Third, an 808-dimensional class vector is used as the input at the second layer. Similarly, the second layer produces an eight-dimensional class vector, which will be concatenated with the 800-dimensional feature vector. And another 808-dimensional class vector is applied as the input at the third layer. Finally, when training on a new layer, a training set is used to tune the parameters and a validation set is utilized to evaluate the performance. The feature importance will be evaluated through the prediction difference between the original LPI features and the learned ones in the four different types of classifiers. The training process will be terminated when the performance is not significantly improved. After training, LPI features with zero importance values are removed and the features with valid importance values are kept. For a test example (an LPI feature), it will be represented by each level until the last level.

**Figure 5:**
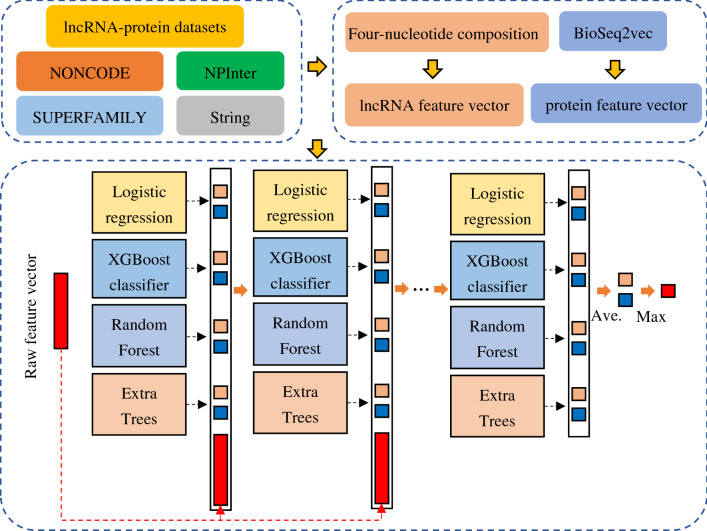
Flowchart of the LPI prediction framework based on deep forest with cascade forest structure.

Figure 5 demonstrates the pipeline of LPIDF. First, five LPI datasets are obtained based on the existing resources. Second, for an lncRNA–protein pair, lncRNA and protein sequences are characterized and concatenated as a vector based on four-nucleotide composition and BioSeq2vec with encoder–decoder structure. Third, the concatenated vector is used as the input to the cascade forest. Finally, the most important features are selected based on layer-to-layer propagation and label of each lncRNA–protein pair is computed.

## Data Availability

Source codes and datasets are freely available for download at https://github.com/plhhnu/LPIDF.
